# Merlin: A Vision Language Foundation Model for 3D Computed Tomography

**DOI:** 10.21203/rs.3.rs-4546309/v1

**Published:** 2024-06-28

**Authors:** Louis Blankemeier, Joseph Paul Cohen, Ashwin Kumar, Dave Van Veen, Syed Jamal Safdar Gardezi, Magdalini Paschali, Zhihong Chen, Jean-Benoit Delbrouck, Eduardo Reis, Cesar Truyts, Christian Bluethgen, Malte Engmann Kjeldskov Jensen, Sophie Ostmeier, Maya Varma, Jeya Maria Jose Valanarasu, Zhongnan Fang, Zepeng Huo, Zaid Nabulsi, Diego Ardila, Wei-Hung Weng, Edson Amaro, Neera Ahuja, Jason Fries, Nigam H. Shah, Andrew Johnston, Robert D. Boutin, Andrew Wentland, Curtis P. Langlotz, Jason Hom, Sergios Gatidis, Akshay S. Chaudhari

**Affiliations:** 1 Department of Electrical Engineering, Stanford University; 2 Stanford Center for Artificial Intelligence in Medicine and Imaging, Stanford University; 3 Department of Radiology, Stanford University; 4 Department of Radiology, University of Wisconsin-Madison; 5 Department of Radiology, Hospital Israelita Albert Einstein; 6 Department of Radiology, University Hospital Zurich; 7 Department of Computer Science, Stanford University; 8 Department of Biomedical Data Science, Stanford University; 9 Department of Medicine, Stanford University

## Abstract

Over 85 million computed tomography (CT) scans are performed annually in the US, of which approximately one quarter focus on the abdomen. Given the current shortage of both general and specialized radiologists, there is a large impetus to use artificial intelligence to alleviate the burden of interpreting these complex imaging studies while simultaneously using the images to extract novel physiological insights. Prior state-of-the-art approaches for automated medical image interpretation leverage vision language models (VLMs) that utilize both the image and the corresponding textual radiology reports. However, current medical VLMs are generally limited to 2D images and short reports. To overcome these shortcomings for abdominal CT interpretation, we introduce *Merlin* - a 3D VLM that leverages both structured electronic health records (EHR) and unstructured radiology reports for pretraining without requiring additional manual annotations. We train Merlin using a high-quality clinical dataset of paired CT scans (6+ million images from 15,331 CTs), EHR diagnosis codes (1.8+ million codes), and radiology reports (6+ million tokens) for training. We comprehensively evaluate Merlin on 6 task types and 752 individual tasks. The non-adapted (off-the-shelf) tasks include zero-shot findings classification (31 findings), phenotype classification (692 phenotypes), and zero-shot cross-modal retrieval (image to findings and image to impressions), while model adapted tasks include 5-year chronic disease prediction (6 diseases), radiology report generation, and 3D semantic segmentation (20 organs). We perform internal validation on a test set of 5,137 CTs, and external validation on 7,000 clinical CTs and on two public CT datasets (VerSe, TotalSegmentator). Beyond these clinically-relevant evaluations, we assess the efficacy of various network architectures and training strategies to depict that Merlin has favorable performance to existing task-specific baselines. We derive data scaling laws to empirically assess training data needs for requisite downstream task performance. Furthermore, unlike conventional VLMs that require hundreds of GPUs for training, we perform all training on a single GPU. This computationally efficient design can help democratize foundation model training, especially for health systems with compute constraints. We plan to release our trained models, code, and dataset, pending manual removal of all protected health information.

Over 85 million computed tomography (CT) scans are performed per year in the US,^1,2,3^ with approximately 300 million CTs performed globally.^4^ Amongst these studies, CTs of the abdomen represent approximately one fourth of all examinations performed.^5^ These abdominal CT scans can consist of upwards of 300 slices per series with numerous anatomical structures that need to be examined, leading to time-consuming interpretation, often requiring 20 minutes per exam.^6^ Moreover, recent literature suggests that abdominal CT scans contain biomarkers of early diseases that routinely go unreported.^7,8,9,10,11,12,13,14^ With the current volume of medical imaging and a 6% annual^15^ increase in medical imaging utilization, the burden on radiologists is significant. Nonetheless, over the past decades, the number of radiology residency positions in the US has remained relatively constant at 1011 in 2006 and 1113 in 2020.^16^ This is despite 1,800+ open radiologist positions on the American College of Radiology job board^17^ from a pool of roughly 32,000 total active radiologists in the US^18^*. With the supply of new radiologists remaining relatively constant and an ever increasing utilization of medical imaging, the radiologist shortage is projected to expand to over 19,000 by 2036.^19,20,21,22^

Machine learning (ML) has shown promise in various medical imaging tasks,^23,24,25,26^ inspiring optimism about its potential to offset the increasing burden faced by radiologists.^19,27^ As of May 2024, of the 882 FDA-cleared ML-enabled devices, 671 (76%) relate to radiology.^28^ Despite this large prevalence, the current status-quo of training ML algorithms entails using unimodal (imaging-only) algorithms and retraining for new tasks from scratch using supervised ML with manually curated labels, even if it may be for the same modality or anatomy. In the medical imaging scenario, generating such labels requires expensive clinical expert time, limiting the development of capable AI models for a wide array of tasks.

Recent years have witnessed remarkable advancements in vision-language models (VLMs), a data-efficient alternative to supervised training. The contrastive language-image pretraining (CLIP) technique^29^ demonstrated the efficacy of aligning text and visual representations in a shared embedding space as a means of supervising vision models with natural language. This paradigm enables leveraging internet-scale images and captions, demonstrating impressive image understanding capabilities in off-the-shelf (zero shot) settings or in settings that use subsequent adaptation (few shot learning)^30^ for a large number of downstream tasks. Such models, trained on large-scale multi-modal pretraining datasets and enabling adaptation for multiple downstream tasks, are commonly referred to as foundation models.

CLIP-based methods could be readily applied in the clinical setting by training with medical images and corresponding radiology reports that are generated routinely during clinical care, adding no additional data labeling cost. Many institutions, including ours, de-identify this data to maintain patient privacy while providing a unique setting for research on this high-quality human-annotated data. Recent ethical viewpoints describe how large-scale already-acquired clinical data could be responsibly used for secondary purposes such as training ML algorithms, *to ensure that the data are used for the benefit of future patients and society*^31^. Following such frameworks, VLMs like MedCLIP,^32^ BiomedCLIP,^33^ LLaVA-Med,^34^ Med-Flamingo,^35^ Med-PaLM M,^36^ RadFM,^37^ XrayGPT,^38^ ELIXR,^39^ RoentGen,^40,41^ CheXagent,^42^ and MAIRA-1^43^ demonstrate the potential of VLMs applied within the radiology domain.

Despite the burgeoning popularity of VLMs for radiology, most existing models focus on 2D modalities such as radiographs, notwithstanding that most medical imaging studies are 3D in nature^†^. Many approaches extend 2D models to 3D by aggregating predictions slice-by-slice or in chunks of slices across the 2D image stacks that make up the 3D volume^44,45^ - an inefficient technique to parse the full 3D imaging volume. Unlike analysis of video, where successive frames have high correlation, there is limited 3D correlation in volumetric anatomical structures that rapidly change in all dimensions. This scenario is well-suited for 3D modeling where features spanning all 3 dimensions are synthesized to generate insights. Moreover, existing methods do not leverage supervision from the multiple data types, including EHR diagnosis codes and radiology reports, available in clinical settings.

The potential clinical impact of 3D medical imaging models may be significant. 3D medical VLMs could assist radiologists by flagging missed findings,^21^ accelerating image interpretation workflows, and serving as AI assistants that draft radiology reports.^46,27^ The opportunity is particularly substantial for abdominal CT exams, which are the most commonly utilized 3D examination^5^ and require significant time for interpretation due to the number of anatomical structures that need to be examined.^6^

Nonetheless, adapting existing methods for training VLMs to 3D medical imaging presents a challenge as a single volumetric image can comprise upwards of 300 individual 2D images, and the corresponding radiology reports can exceed 300 words.^6^ Training models on these large data samples can also require significant computational resources, which are often not available in academic institutions or within hospital systems where the data resides.^47^ Furthermore, clinically-relevant evaluations for benchmarking 3D VLMs across a suite of tasks is still lacking. Even in the clinical large language model (LLM) space, where progress is rapid, popular benchmarks based on medical licensing exams garner criticism for not reflecting real-world clinical use cases.^48,49^ Due to these training and evaluation challenges, there exists a dearth of methods for training and evaluating 3D medical imaging VLMs that can be adapted for a wide range of downstream tasks.

In this paper, we focus on developing and evaluating foundation VLMs for 3D abdominal CT scans to enhance image understanding across a variety of tasks. Our work provides the following contributions:

1. We develop a training strategy and foundation model called *Merlin*^‡^ that leverages the structured and unstructured data within hospitals to train an abdominal CT visual model. Using this training strategy, we train an extremely compute-efficient vision model on a single GPU using paired CTs (6,387,231 images from 15,331 CTs), EHR diagnosis codes (1,839,559 codes), and radiology reports (6,036,645 tokens). Merlin allows processing the entire 3D voxel data in a CT image at once.
2. We evaluate Merlin on a comprehensive set of 6 task types and 752 individual tasks. The non-adapted (off-the-shelf tasks) include zero-shot findings classification (31 findings), phenotype classification (692 phenotypes), and zero-shot cross-modal retrieval (image to findings and image to impressions), while model adapted tasks include 5-year disease prediction (6 diseases), radiology report generation, and 3D semantic segmentation (20 organs).
3. We perform internal validation on 5,137 CTs as well as external validation on 7,000 CTs from another institution, and on two publicly available datasets focused on abdominal CT (VerSe^50^ and TotalSegmentator^51^). We demonstrate that our singular Merlin model outperforms carefully chosen task-specific baselines on our suite of benchmarking tasks.
4. We derive data scaling laws for Merlin, providing guidance about the data requirements for achieving specific levels of performance. We also present comprehensive ablation studies demonstrating the impact of various training strategies and the role of the different clinical data types on model performance.
5. We plan to release our trained models, code, and dataset, pending manual review and removal of all personal health information.

## 2 Results

**Figure 1:**
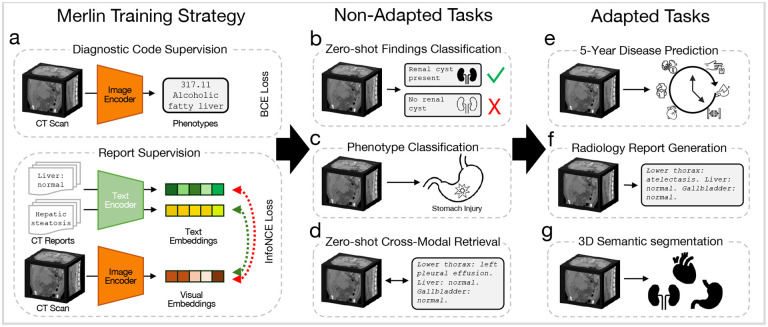
Overview of Merlin training and evaluation. (a) Merlin training strategy. Diagnosis codes from the EHR are used as labels for Merlin training, with a binary cross entropy loss. Radiology reports are also used for training, with an InfoNCE loss.^52^ Training with diagnosis codes and radiology reports is either staged or performed in a multi-task manner. Merlin is then evaluated on non-adapted tasks that can be performed without any architectural or weight modifications. These include (b) zero-shot findings classification, (c) phenotype classification, and (d) zero-shot cross-modal retrieval. Adapting Merlin enables us to perform (e) 5-year disease prediction, (f) radiology report generation, and (g) 3D semantic segmentation. All error bars are 95% confidence intervals.

We present results across 6 evaluation task types comprising 752 individual tasks, that can be performed of-the-shelf without adaptation (Figure 1b - Figure 1d), or with additional adaptation (Figure 1e - Figure 1g). The non-adapted tasks that we evaluate Merlin on include zero-shot classification (31 classification tasks; Figure 1b), phenotype classification (692 classification tasks; Figure 1c), and zero-shot cross-modal retrieval (image to findings and image to impressions; Figure 1d). The adapted tasks that we evaluate Merlin on include 5-year disease prediction (6 classification tasks; Figure 1e), radiology report generation (Figure 1f), and 3D segmentation (20 organs; Figure 1g).

### 2.1 Zero-shot Findings Classification

**Figure 2:**
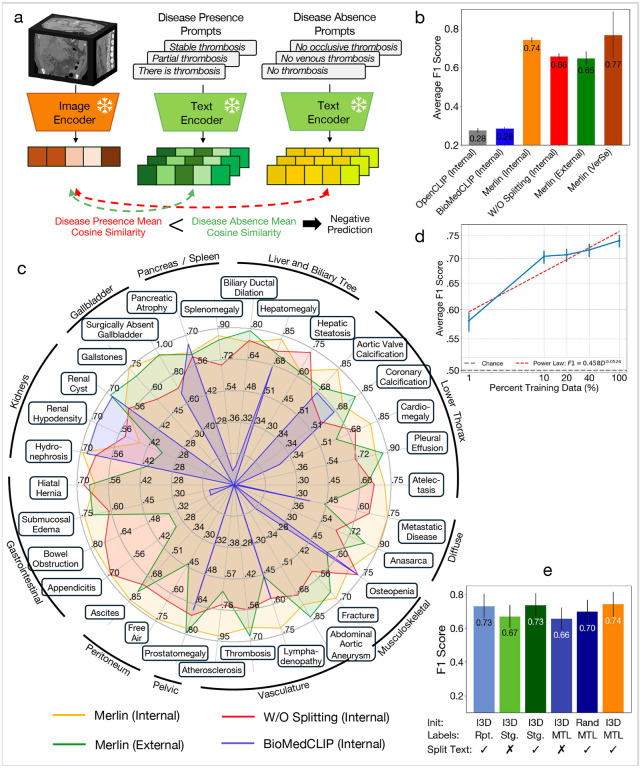
Zero-shot findings classification. (a) Depicts how zero-shot classification is performed where text embeddings from disease presence prompts and disease absence prompts are compare to the image embedding. (b) We compare the performance of OpenCLIP,^53^ BioMedCLIP,^54^ Merlin on an internal dataset, and Merlin without radiology report splitting. We further evaluate Merlin on an external clinical dataset and the VerSe^55^ external fracture detection dataset. (c) Performance of BioMedCLIP, Merlin, and Merlin without report splitting on the internal dataset, as well as Merlin on the external dataset, across 30 findings assessed on abdominal CT scans. (d) Merlin zero-shot classification performance improves with increasing pretraining dataset size. (e) An ablation study across various aspects of Merlin’s pretraining strategy. “Rpt.” is shorthand for “report” and indicated training with radiology reports only. Staged (Stg.) refers to performing weakly supervised training with EHR in a first training stage and then training with radiology reports in a second stage. This is in contrast to multi-task learning (MTL) where EHR and radiology reports are used for training simultaneously.

Zero-shot findings classification assesses Merlin’s ability to classify the presence of common imaging findings based on text prompts that a user can develop, which are likely distinct from prompts seen during training. In Figure 2b we evaluate Merlin zero-shot classification across 30 abdominal CT findings on our internal and external clinical datasets. Merlin achieves an average F1 score of 0.741 (95%CI [0.727–0.755]) on the internal validation dataset and an average F1 score of 0.647 (95%CI [0.607–0.678]) on the external validation dataset, significantly outperforming OpenCLIP^53^ and BioMedCLIP^54^ (p < 0.001) in both settings (Figure 2b). Qualitatively, we observe in Figure 2c that Merlin’s external performance remains high on diseases with coarse-grained or salient features, e.g. pleural effusion, splenomegaly, ascites, surgically absent gallbladder, prostatomegaly, anasarca, and abdominal aortic aneurysm. Performance expectedly decreases on more challenging findings that require more subtle and fine-grained features, e.g. appendicitis, metastatic disease, lymphadenopathy, and free air. We also plot Merlin performance without radiology report splitting (W/O splitting in Figure 2c). Radiology report splitting refers to splitting the radiology reports into sections describing different anatomical structures for subsequent contrastive learning (e.g. *“liver: normal”* and *“vasculature: patent”*). Without radiology report splitting, Merlin achieves an average F1 score of 0.656 (95%CI [0.640, 0.671]), similar to the Merlin external evaluation performance. On a separate external dataset (VerSe^55^ vertebral fracture detection), Merlin achieves a zero-shot F1 of 0.767 (95%CI [0.623–0.867]). In Figure 2d, we establish quantitative relationships to assess how expanding the pretraining dataset would improve zero-shot classification. This analysis helps determine the extent of pretraining data necessary for obtaining a specified zero-shot classification performance. The resulting power law is $\overset{-}{F1}={0.458D}^{0.0524}$, with *D* representing the number of paired pretraining CT imaging and report data.

Compared to the ablations, Merlin (I3D initialization^56^ where 2D ImageNet pretrained weights are reused within the 3D model, multi-task learning with EHR and radiology reports versus training in stages, and radiology report splitting) performs the best with an F1 score of 0.741 (95%CI [0.727–0.755]) (Figure 2e). Report splitting and staging training across the EHR and radiology reports results in second best performance. Learning directly from the radiology reports without any EHR supervision, along with radiology report splitting, results in the third best performance. These settings achieve F1 scores of 0.735 (95%CI [0.719–0.748]) and 0.730 (95%CI [0.714, 0.744]), respectively. The largest impact on performance is driven by the choice of whether or not to split the radiology reports. Relative to Merlin, without report splitting, the performance drops by an average of 7.9 F1 points (p « 0.01).

### 2.2 Phenotype Classification

The goal of this task is to use CT scans to directly predict phenotypes^57^ (based on groupings of ICD codes) that were assigned to patients during their hospital admission that included the CT scan. We evaluate the performance of Merlin in predicting 692 phenotypes defined in PheWAS.^57^ We find that Merlin reaches a macro-average area under the receiver operating characteristic curve (AUROC) across phenotypes of 0.812 (95% CI, 0.808–0.816), and achieves AUROCs over 0.85 for 258 phenotypes (37% of all phenotypes), and AUROCs over 0.9 for 102 phenotypes (15% of all phenotypes).

**Figure 3:**
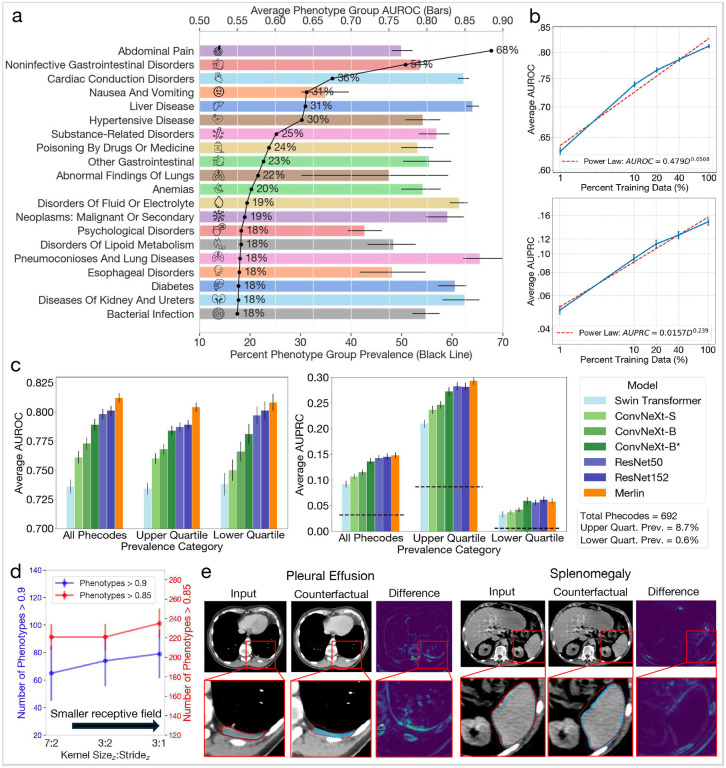
Phenotype classification. (a) Average AUROC performance for the top 20 phenotype groups listed in order of prevalence (black line). (b) Data scaling law experiments that measure how average AUROC (top) and average AUPRC (bottom) across the 692 phenotypes scale as the amount of pretraining data varies. (c) Average AUROC (left chart) and AUPRC (right chart) across all 692 phenotypes, the top quartile of 173 phenotypes, and the botton quartile of 173 phenotypes across several baseline models. All baseline models are trained using the phenotypes in the pretraining dataset. The dashed lines denote random chance performance. Note that Merlin, which uses the best performing backbone of ResNet152, is further trained using radiology reports. (d) Average AUROC as a function of model stem hyper-parameters. We find that a smaller receptive field yields better performance. (e) Counterfactual analyses of pleural effusion classification (left; image from TCIA^61^) and splenomegaly classification (image from our internal test set). We annotate the zoomed in images by outlining the pathologies. The red lines border pathologies in the original images. The blue lines border pathologies in the counterfactual images. Counterfactual outlines are drawn over the original images with dotted lines and the original image outlines are also drawn over the counterfactual images with dotted lines. This allows comparing the size and shape of the pathologies between the original images and the counterfactuals, indicating that Merlin is indeed using appropriate features for image classification.

In Figure 3a, we group the 692 phenotypes into groups of similar phenotypes using established methods (exclusion ranges defined in the PheWAS database^57^) and report prevalence and average performance within the top 20 most prevalent groups in the internal test set. We find that abdominal pain is the most prevalent phenotype group (68% prevalence), followed by noninfective gastrointestinal disorders (51% prevalence). Measuring average model performance across the phenotypes within each group, we find that Merlin performs well in detecting diseases across a range of organ systems, including the liver, kidneys, ureters, and gastrointestinal tract.

In Figure 3b we compute data scaling law curves to assess how Merlin performance improves with respect to training data. Similar to zero-shot classification, we find that increasing training data improves performance.Our analysis also reveals power laws with the following slopes and intercepts: ($\overset{-}{\text{AUROC}}={0.479D}^{0.0568}$ and $\overset{-}{\text{AUPRC}}=0.0157^{x0.239}$).

In Figure 3c and Figure 3d, we compare performance across 7 model variations, with different model backbones, architectures, and training paradigms. We make the following observations: i) model performance consistently improves across model size, ii) smaller convolutional receptive fields improve performance, with smaller z-dimension kernel sizes and strides in the model stem performing best (Figure 3d), iii) ResNet backbones, with in-plane and out-of-plane kernel size of 3, outperform ConvNeXt^58^ backbones (in-plane kernel size of 7) that have out-of-plane kernel sizes of 3 (ConvNeXt-B*) or 7 (ConvNeXt-B) at all layers of the network (Figure 3c). The Swin Transformer^59^ backbone performs the worst, with a window size of 7 and patch size of 4. Due to the computational complexity of the attention mechanism, the number of parameters of the Swin Transformer is lowest (3 million). The trend that we observe across model variations as measured by average AUROC aligns with the trend described by average AUPRC.

In Figure 3e, we apply the latent shift counterfactual method^60^ to qualitatively investigate the image features that Merlin uses to perform image classification. We examine an instance of “pleural effusion” (left) where the effusion is localized to the left lung and is reduced in the counterfactual, indicating that Merlin is leveraging the features we expect. We also examine “splenomegaly” classification (right). We observe that the size of the spleen is reduced in the counterfactual relative to the original image, adding credence to the validity of imaging features that Merlin uses for image classification.

### 2.3 Zero-shot Cross-Modal Retrieval

**Figure 4:**
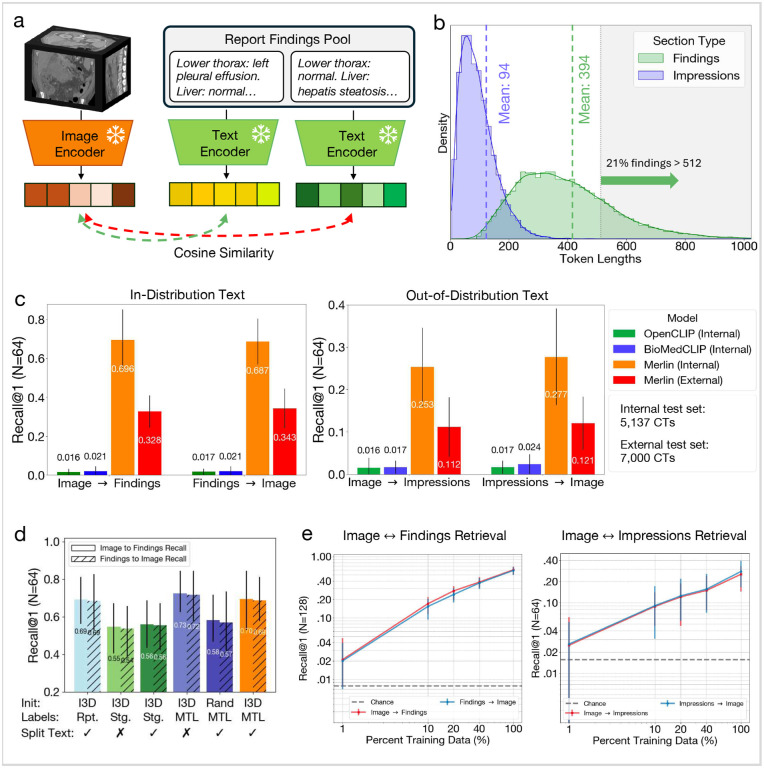
Zero-shot cross-modal retrieval. (a) Schematic demonstrating how we perform retrieval. We compute the cosine similarity between Merlin report embeddings and CT embeddings, enabling us to rank CT and report pairs in order of similarity. (b) A distribution of the findings section and impressions section lengths shows that 21% of findings have sequence lengths greater than 512 tokens. (c) Top-1 recall out of pools of 64 findings sections (left), which is considered an in-distribution evaluation as Merlin is trained using findings sections. We also report top-1 recall on out-of-distribution impressions sections (right). (d) An ablation study that examines the impact of using I3D ImageNet initialization, multi-task learning (MTL) versus staged training (Stg.) with EHR and reports versus training with reports only (Rpt.), and splitting the radiology report text into anatomical sections. (e) Data scaling law experiments that examine the impact of pretraining dataset size on retrieval performance. The dashed lines indicate random chance performance.

Zero-shot cross-modal retrieval evaluates the model’s ability to match a CT image with the corresponding radiology report findings or impressions section and vice versa. In Figure 4b, we plot the distribution of radiology report findings and impressions lengths. We find that 21% of findings sections have lengths that exceed 512 tokens, motivating our choice of the clinical Longformer^62^ text encoder (OpenCLIP^53^ and BioMedCLIP^54^ only allow maximum token lengths of 77 and 256, respectively). We find that Merlin significantly (p << 0.01) outperforms OpenCLIP and BioMedCLIP on the task of 3D retrieval for retrieving the correct findings out of 64 cases (Image → Findings in Figure 4c). We find similar performance for retrieving the correct CT scan out of 64, given a findings section (Findings → Image in Figure 4c).

Retrieving the impressions section from radiology reports provides evidence that Merlin generalizes to out-ofdistribution text, given that Merlin training uses the findings sections. Figure 4c demonstrates that Merlin generalizes to the task of retrieving the correct impressions section given a scan (Image → Impressions) and retrieving the correct scan given an impressions section (Impressions → Image). On the external test set, retrieval performances decreases (Figure 4c). However, it is important to note that the structure and language used in reports varies significantly across institutions. Nonetheless, the external Merlin performance remains 5–7x better than the external baselines.

We conduct a model ablation study (Figure 4d) to investigate i) the impact of ImageNet initialization with I3D weights, ii) the impact of either staged training or multi-task learning using the EHR and radiology reports, and iii) the impact of training with full reports or reports that are split across anatomical regions. We find that the top-performing model variation uses I3D initialization, multi-task learning with the EHR and radiology reports, and no report splitting. We hypothesize that report splitting does not improve performance on the retrieval task since retrieval prompting is performed with the full report sections. Splitting the report into subparts and additionally learning directly from the radiology reports without any EHR supervision results in the second and third best performance, respectively.

Finally, we find that model performance improves with pretraining dataset size both for image and findings retrieval and image and impressions retrieval via the data scaling curve in Figure 4e.

### 2.4 Multi-Disease 5-Year Prediction

**Figure 5:**
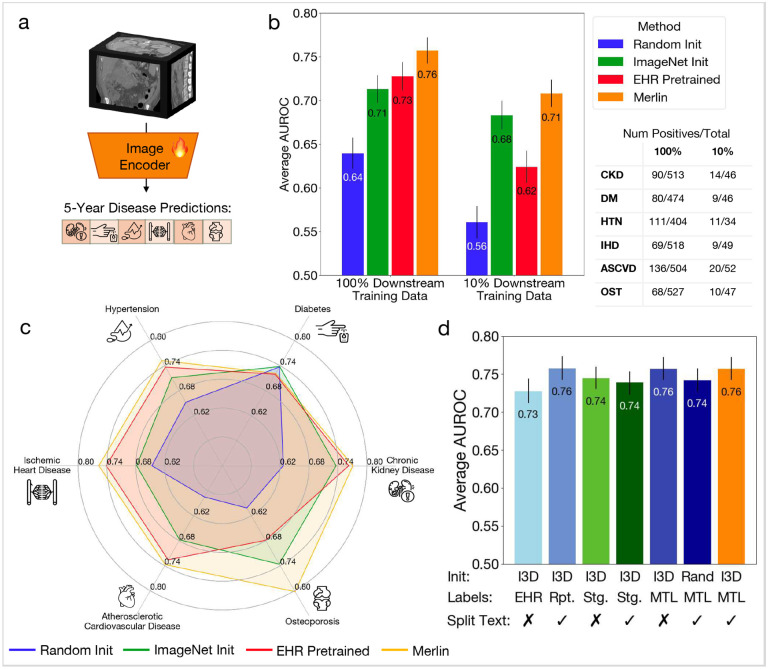
Multi-disease 5-year prediction. (a) We fine-tune Merlin for predicting chronic disease onset in otherwise healthy patients within 5-years. (b) We compare Merlin to other baseline model variations fine-tuned for the same task. We find that with both 100% and 10% of downstream training data, Merlin outperforms the other model variations. (c) Comparison of Merlin chronic disease prediction performance to a model trained using only phenotypes (EHR Pretraining), an ImageNet I3D initialized model, and a randomly initialized model. (d) An ablation study that measures the impact of various aspects of Merlin’s training strategy. We find that training with EHR and radiology reports, using staged training (Stg.) or multi-task learning (MTL), and training with radiology reports only (Rpt.), all outperform training with EHR only.

Multi-disease 5-year prediction measures the model’s ability to predict whether a patient will develop a chronic disease within 5 years, given that they are not diagnosed with the disease when their CT scan was acquired. We fine-tune Merlin to opportunistically predict which patients, who are healthy at baseline, will be diagnosed with any of 6 chronic diseases (chronic kidney disease, osteoporosis, cardiovascular disease, ischemic heart disease, hypertension, and diabetes) in the ensuing 5 years based on their CT scan. On average across these 6 diseases, Merlin predicts disease onset within 5 years with an AUROC of 0.757 (95%CI [0.743, 0.772]) using 100% of the downstream labels, and outperforms the ImageNet pretrained (I3D) image-only model by 7% (Figure 5b). Even using 10% of labels, Merlin predicts disease onset within 5 years with an AUROC of 0.708 (95%CI [0.692, 0.723]), and outperforms the ImageNet pretrained model by 4.4% (Figure 5b). Using Merlin for disease risk stratification can produce similar accuracy as using an ImageNet pretrained model, while utilizing 10x reduced labeled training data. These results depict that even fewer than 25 positive cases (10% of training data) can be used to build future disease risk stratification models using Merlin. Our model ablation study demonstrates that 3 configurations, all of which use I3D initialization, provide similar performance. Report training only with report splitting (AUROC of 0.758 (95%CI [0.743, 0.773])), multi-task EHR and report training without report splitting (AUROC of 0.757 (95%CI [0.743, 0.772])), and our Merlin configuration with multi-task EHR and report training, along with report splitting, (AUROC of 0.757 (95%CI [0.743, 0.772])) all produce comparable results.

### 2.5 Radiology Report Generation

Radiology report generation evaluates Merlin’s capacity for generating reports based on the CT images. We select RadFM^37^ as a baseline, as it is a multi-modal text generation model where the training dataset includes abdominal CT scans. Based on the quantitative metrics of RadGraph-F1,^63^ BERT Score,^64^ ROUGE-2,^65^ and BLEU score,^66^ Merlin consistently outperforms RadFM across anatomical sections and the full report findings (Figure 8b).

Qualitatively, we observe that Merlin generates reports with correct structure, where findings are placed within the correct anatomical section. When Merlin predicts the presence of a finding, the finding or a related finding usually exists in the image. For example, in the Merlin generated report in Figure 8c, Merlin correctly identifies that “the endometrial strip is thickened”. Nonetheless, we observe that Merlin tends to under-report positive findings. For instance, in the example in Figure 8c, Merlin does not report the cholelithiasis finding, which is present in the human generated report and in the CT image. As this is an early demonstration of radiology report generation based on CT scans, we anticipate room for improvement in the generated reports.

### 2.6 3D Semantic Segmentation

**Figure 7:**
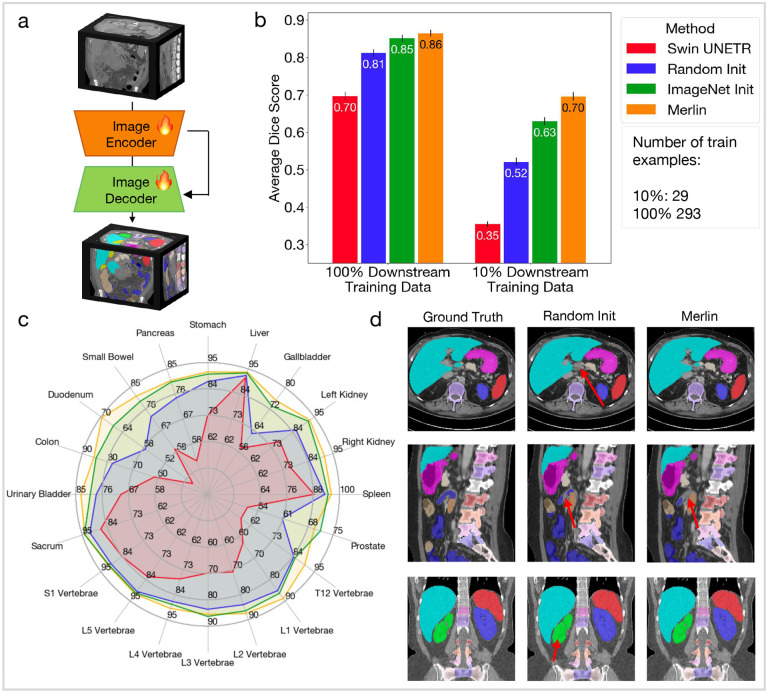
3D semantic segmentation. (a) To adapt Merlin for segmentation, we add a decoder and skip connections between the Merlin encoder and decoder. (b) We compare model variations using average Dice score across 20 organs that appear in abdominal CT. We compare performance of models trained using 100% of training cases and also simulate the data scarce regime with 10% of training cases. (c) We report Dice scores for 20 organs across 4 model variations using 100% of training cases. (d) We qualitatively compare segmentations between the ground truth labels, a model with the Merlin architecture that has been randomly initialized, and Merlin. The red arrows indicate mistakes made by the model relative to the ground truth. Each row is a different patient sampled from the Total Segmentator^51^ test set.

3D semantic segmentation evaluates whether Merlin learns geometric information about various anatomical structures. We find that with 10% of training cases, Merlin performs 11% better than the second best model variation, which is the Merlin architecture with ImageNet I3D initialization (Figure 7b). This demonstrates that Merlin pretraining is particularly beneficial in label scarce scenarios. Although the advantage is reduced with 100% of training labels, Merlin still outperforms the other model variations (Figure 7b). Figure 7c shows that, where performance of baseline models tends to saturate for larger organs (e.g. liver, stomach, and spleen), Merlin performs best relative to baselines on smaller organs or organs with complicated shapes, like the duodenum, small bowel, T12 vertebrae, L1 vertebrae, and gallbladder. Figure 7d presents qualitative segmentation results, where the red arrows indicate differences in the predicted segmentations relative to the ground truth. We observe that the randomly initialized model makes minor mistakes in all three scans, while Merlin makes a mistake in one of the three scans. Top row, center column: the randomly initialized model misses a small part of the caudate liver lobe. Middle row, center and right columns: both Merlin and randomly initialized models miss small portions of the proximal jejunum. Bottom row, center column: the randomly initialized model includes the bottom calyces of the right kidney in the segmentation mask.

## 3 Discussion

In this study, we curate a high-quality clinical dataset comprising of paired CT scans (10,628,509 total 2D images from 25,528 CT scans), EHR data (2,995,293 ICD codes), and radiology reports (10,051,571 total tokens in the findings sections). With this dataset, we train Merlin, a 3D vision-language foundation model for interpreting abdominal CT scans that leverages structured EHR data and unstructured radiology report supervision. Merlin is trained on a single GPU, demonstrating how hospitals and research institutions can pragmatically build their own models, as well as highlighting the opportunity to efficiently train larger models with additional compute and data. Despite the minimal resources used to train Merlin, we demonstrate that Merlin can generalize to 6 types of downstream tasks with 752 individual tasks. Non-adapted tasks include zero-shot findings classification (31 classes), phenotype classification (692 classes), and zero-shot cross-modal retrieval (including both image to findings and image to impressions retrieval). Model adapted tasks include 5-year disease prediction (6 classes), radiology report generation, and 3D semantic segmentation (20 organs). We outperform carefully chosen single-task baseline models across all tasks. We further contextualize our methods with comprehensive ablation studies that demonstrate Merlin’s performance as a function of the underlying model architecture and the design considerations for the use of the underlying data. For training our Merlin foundation model, we demonstrate the benefit of I3D weight initialization, multi-task learning with EHR and reports, and splitting the radiology reports into anatomical sections. Overall, we depict how Merlin may assist in interpretation of abdominal CT scans and mitigate the burden on radiologists, while simultaneously adding value for new biomarker discovery and future disease risk stratification using pre-existing datasets.

Prior approaches for 3D CT pretraining focus on image-only pretraining.^67,68,69^ These methods leverage masked autoencoders (MAEs),^70^ which train a model to reconstruct the input image, given that some part of the input is masked out. This approach has been demonstrated to be beneficial for 3D segmentation tasks, where the decoder network is included in pretraining. However, the quality of the latent space representations for a variety of downstream tasks on CT images has not been demonstrated. It is possible that while MAEs require the latent space to encode the geometric information necessary to reconstruct the input, this geometric information reduces the effectiveness of the latent representations for image classification tasks. For image classification, it may be beneficial to discard this geometric information. In contrast to MAEs, Merlin trains the latent representations directly using supervision from EHR diagnosis codes and radiology reports. We demonstrate that these representations are useful for a variety of downstream task types, including tasks involving text and images, which cannot be accomplished using image-only pretraining methods.

Recent concurrent research has started to investigate 3D vision language models for radiology, particularly focusing on chest and head CT scans.^71,72^ However, the extent of experiments and 3D evaluations are limited. These studies lack systematic ablation studies and do not utilize all available clinical data, including EHR. In addition to exploring the use of multiple clinical data types for supervision, we carry out a wide array of experiments and evaluations that contextualize the methodological choices we make in the development of Merlin.

Through the ablation studies, we find that I3D weight initialization^56^ is helpful for all tasks that we examine with ablation studies. Furthermore, training with both EHR diagnoses and radiology reports is beneficial over training with either alone. The manner of combining these supervision sources is important, with multi-task training using EHR and radiology reports outperforming staged training. Additionally, we find that report splitting, while essential for zero-shot classification, slightly degrades zero-shot retrieval performance. This reinforces that pretraining is most effective when the pretraining data distribution matches the downstream task distribution. Splitting the radiology reports results in text that better aligns with prompts for zero-shot classification, but less closely resembles the radiology report text used for retrieval tasks.

In light of our findings, we identify key areas that could significantly enhance the performance of Merlin and other future 3D medical imaging models:

1. *Increasing dataset size:* Expectedly, our initial data scaling results show that using a larger pretraining dataset improves Merlin’s performance (Figure 3b, Figure 4e, Figure 2d). Our data scaling curves across tasks can serve as a useful prospective reference measure in evaluating adequate sizes of training datasets for a requisite task performance. We note that as we add more data, we may need to update our model architecture, which was optimized at our current data scale.
2. *Improving image resolution:* Utilizing higher resolution images is expected to enhance model performance, as suggested by prior studies on vision-language models.^73,74,75^ This may only hold until the resolution used for training is equal to the resolution of the original CT scan, and we are no longer downsampling the CT scan. It is important to note that increasing the physical resolution during image acquisition may not always yield better results. Radiologists often do not use the highest resolution available during acquisition in abdominal CT scans, particularly regarding slice thickness, since this may come at the expense of lower signal-to-noise ratio (SNR).
3. *Optimizing batch size:* Improving batch size is a challenge with 3D medical imaging where the size of the images is significant. However, prior work demonstrates that large batch sizes are beneficial for contrastive pretraining.^75,76^
4. *Extending to additional anatomies and modalities:* Our Merlin model lays the groundwork for training anatomy- and modality-specific radiology foundation models. Future work can explore the relative benefits of pretraining on multiple anatomies for the same modality, multiple modalities for the same anatomy, or both. For such experiments, it is imperative to benchmark the benefit that each modality or anatomy provides, and we hope that our rigorous evaluation strategy can help highlight optimal data mixtures for training next-generation radiology foundation models.

Beyond the strengths of our study, there are also limitations of our model and our work. First, towards our goal of democratization of foundation models, we train and evaluate Merlin using a single GPU. This may limit the generalizability of some of our findings as task performance may increase by simply scaling up compute resources. Second, the limited number of publicly-available baselines for abdominal CT, especially VLM baselines, makes it challenging to understand the relative efficacy of our methods. On the other hand, we believe that Merlin could establish a strong and task-agnostic initial baseline for abdominal CT. Third, further exploration is required to optimize Merlin for the tasks of radiology report generation and 3D segmentation. For radiology report generation, there are numerous parameters that can be tuned, primarily regarding the adapter and LLM. While we devise an initial performant strategy for direct report generation, we leave further optimization of the adapter and LLM to future work. Likewise, adapting Merlin for segmentation requires adding a decoder network. Future work can explore additional architectural parameters and optimisation strategies for decoder training.

## 4 Methods

### 4.1 Datasets: Paired CT Scan, Unstructured Radiology Report, and Structured EHR

Medical imaging presents the opportunity to capture supervision signal from both the structured information in the EHR, as well as the unstructured information in radiology reports. We focus on abdominal CT images, as abdominal CTs are the most common 3D imaging examination.^5^

All datasets used in this study were under IRB approval with a waiver of informed consent due to the use of retrospective data. To collect the dataset, we identified patients from our academic medical center who underwent consecutive abdominal CT examinations from December 2012 to October 2018. This resulted in a high quality clinical dataset comprising 18,321 patients, from inpatient (37%), emergency services (35%), outpatient (16%), and observation (8%). We collected the following data for each patient:

#### CT Studies:

We obtain full abdominal CT studies, each comprising multiple CT series. From each study, we select the series with the most axial slices, maximizing the amount of information in the CT volume. This results in 10,628,509 2D images from 25,528 CTs. This sampling may include the non-contrast series and put the image embeddings at a disadvantage relative to the radiology reports since contrast enhancement patterns may not be apparent on the non-contrast series. To remedy this, we run an open source abdominal CT contrast-phase detection algorithm that has been validated previously^77^ on the selected CT series. We use this algorithm as the image meta-data related to contrast phase is often missing or inaccurate. We find that 97% of the selected series are portal venous phase, while 2.4%, 0.45%, and 0.26% scans are delayed, arterial, and non-contrast phases, respectively.

#### Radiology Reports:

We compile the associated radiology reports for each CT study. These reports consist of multiple sections, most predominantly the findings and impressions sections. The findings section includes detailed observations from each organ system. The impressions section contains a description of the most clinically important findings. We use only the findings sections for training, given the granularity of information provided and previous work demonstrating the efficacy of this approach.^78^ We find that there are 10,051,571 tokens from the radiology report findings sections in our dataset.

#### EHR:

Beyond the images and reports, we acquire EHR data for each patient. In this work, we leverage the diagnosis information, in the form of ICD codes, for model training. To link ICD codes with CT scans, we collect the ICD codes that were assigned during the patient encounter that generated the CT. There are 954,013 assigned ICD9 codes with 5,686 unique ICD9 code values in our dataset. There are 2,041,280 ICD10 codes with 10,867 unique ICD10 code values. In total, there are 2,995,293 assigned codes in the dataset with 16,553 unique values.

### 4.2 Vision-Language-EHR Model

We aim to leverage both structured EHR and unstructured radiology report information as supervision signal to train a CT visual model.

#### CT Scan Preprocessing:

We reformat all CT scans so that the first axis points from left to right, the second from posterior to anterior, and the third from inferior to superior. We then resample the in-plane axial images to 1.5mm resolution and the out-of-plane slice thickness to 3mm spacing using bilinear interpolation. We map the Hounsfield unit range −1000:1000 to the range 0:1, clipping values that fall outside of this range. Finally, we pad and center crop to 224 by 224 pixels in-plane and 160 pixels out-of-plane.

#### EHR Preprocessing:

From the EHR, we extract all ICD-9 and ICD-10 codes that are assigned during the hospital visits where the abdominal CT studies are carried out. Furthermore, we observe that often codes can comprise disparate characters but have similar underlying phenotypic meaning. For example, the ICD-9 code V12.55 denotes a personal history of pulmonary embolism, where ICD-9 code 415 represents acute pulmonary heart disease. While one of these codes describes a history and one describes an acute indication, their imaging phenotypes may appear similar. Thus, we leverage the PheWAS Phecode Mapping^57^ to map 16,553 ICD-9 and ICD-10 codes to 1,692 hierarchical phenotypes. Furthermore, we apply phenotype expansion where if a subject is positive for a more specific phenotype during a particular hospital visit, we propagate this positive label throughout the hierarchical phenotype tree so they are also positive for all less specific phenotypes. Grouping the ICD codes in our dataset results in 1,692 total phenotypes. For each CT image, we thus have an associated binary vector with a 0 indicating the absence of a phenotype during the corresponding hospital visit and a 1 indicating the presence of the phenotype. This supervision signal is coarse grained in that the phenotypes may not be directly associated with pixel values in the abdominal CT scan; they are associated with the patient’s health status more generally. Thus, the EHR phenotype codes serve as weak supervision for our model training.

#### Radiology Report Preprocessing:

Using regular expressions, we extract the findings section from each radiology report. Due to the long radiology reports, we hypothesize that contrastive training may overfit to short and salient parts of the reports to solve the task. Furthermore, short prompts used for subsequent zero-shot classification would present a significant domain shift compared to the full reports. Thus, we split the reports into anatomical sections and during each training step, alternate between presenting the full reports and a single anatomical section. We rotate through the anatomical sections every other step, presenting the model a single anatomy per batch to allow the model to compare across different descriptions of the same anatomy. To generate these sections, we use regular expressions. If the regular expressions fail for a particular report and anatomy, we use the full report. The sections that we consider are lower thorax, liver and biliary tree, gallbladder, spleen, pancreas, adrenal glands, kidneys and ureters, gastrointestinal tract, peritoneal cavity, pelvic organs, vasculature and lymph nodes, and musculoskeletal.

#### Model Architecture:

Merlin uses an inflated 3D (I3D) ResNet152 for the image encoder. Inflation refers to reusing 2D pretrained model weights and copying those weights across the 3rd dimension of the 3D convolutional kernels.^56^ Given the long token lengths of the reports (Figure 4b), we use clinical Longformer^62^ as the text encoder due to its longer context length (4,096) than other biomedical pretrained masked language models and general domain CLIP text encoders. Previous work found that pretrained text encoders with longer context length perform better, given longer captions.^33^ We also perform architecture ablation studies where we investigate 3D Swin Transformer^59^ and ConvNeXt^58^ architectures (Figure 3c). In addition to architecture ablations, we investigate how the out-of-plane stride and kernel size in the model stem of the ResNet152 impact performance (Figure 3d). The model stem in the ResNet152 is a convolutional layer at the input of the network with an in-plane kernel size of 7 and stride of 2, followed by a max pooling layer with a kernel size of 3 and a stride of 2. The model stem thus reduces size of the input by a factor of 4 in each dimension. The model stem ablation only adjusts parameters in the convolutional layer of the stem. We maintain the in-plane settings as they are in the 2D version of the models to leverage the 2D pretraining. Furthermore, we assess how inflating the ConvNeXt model with an out-of-plane kernel size of 7, matching the 2D kernel size of the original 2D weights, performs compared to inflating the ConvNeXt model with an out-of-plane kernel size of 3. We refer to the model with an out-of-plane kernel size of 3 as ConvNeXt-B* in Figure 3c. Additionally, we compare various model sizes in Figure 3c. We perform these architecture experiments using the phenotype classification task due to the simplicity of this supervised task and the strong evaluation signal that results from averaging performance across a large number of phenotypes.

#### Model Training:

We use binary cross entropy for the phenotype classification loss and InfoNCE^52,29^ loss for contrastive learning with the radiology reports. We use an AdamW^79^ optimizer with an initial learning rate of 1e-5, betas=(0.9, 0.999), and a cosine learning rate scheduler with number of epochs for decay to 0 set to 300. We use gradient checkpointing for both the visual and text encoders and train with FP16 mixed precision. This allows us to maximize a batch size of 18 on a single 48GB A6000 GPU.

#### Multi-Task Learning Versus Staged Training:

In addition to training using the EHR phenotypes and radiology reports jointly in a multi-task manner, we consider staging the training. In this formulation, we first train the Merlin image encoder using the EHR phenotypes in stage 1. In stage 2, we perform contrastive training with the radiology reports. To prevent catastrophic forgetting^80^ of the EHR information learned in stage 1, we include the phenotype loss function during stage 2 training, with a low relative weight. We use the same hyper-parameters for stage 2 as we do for multi-task training. For stage 1, we use an AdamW optimizer with an initial learning rate of 1e-4 and betas=(0.9, 0.999), as well as an exponential learning rate scheduler with gamma = 0.99. We use a batchsize of 22 on a single A6000 GPU.

#### Data Splits:

We divide the pretraining dataset into splits of size 60% (15,331 CTs) for training, 20% (5,060 CTs) for validation, and 20% (5,137 CTs) for testing. We ensure that multiple CTs from a single patient exist in a single split. The external dataset, from another university medical center, consists of 7,000 CTs which are used for testing.

### 4.3 Evaluations

We select evaluation tasks consisting of non-adapted tasks, which Merlin can perform out of the box without additional adaptation. These tasks include zero-shot findings classification, phenotype classification, and zero-shot cross-modal retrieval. We also select tasks that require adapting Merlin, which include 5-year disease prediction, radiology report generation, and 3D semantic segmentation. We select both non-adapted tasks and adapted tasks to demonstrate Merlin’s effectiveness without fine-tuning, as well as its adaptability when fine-tuned for specific applications.

### 4.4 Non-Adapted Tasks

#### Zero-Shot Findings Classification:

We consult three radiologists to develop a list of 30 findings that exhibit a diversity of size and level of difficulty for human diagnosis. For each finding, we generate lists of disease presence phrases that describe possible sub-types, locations, and severities of the finding. We similarly generate a list of disease absence phrases, which are ways of describing a negative finding. For example, a disease presence phrase for ascites could be *“large volume ascites present”* and a disease absence prompt could be *“no ascites”*. We use these phrases to mine the radiology reports for negative and positive examples. To standardize the chance metric value across findings, we balance the number of positive and negative examples for each finding. After extracting these examples, we manually review them to ensure that the labels are accurate.

To perform zero-shot classification, we follow the procedure in Figure 2a where we embed the CT scan using the image encoder. We use the disease presence and absence phrases as prompts for zero-shot classification. We embed each of the prompts using the text encoder. We then compute the cosine similarity between the CT scan embedding and each of the prompts. Computing the mean cosine similarity across the disease presence prompts and mean similarity across the disease absence prompts allows us to classify the CT scan as positive or negative for a given finding. We adapt existing 2D baselines (OpenCLIP^53^ and BioMedCLIP^54^) for 3D zero-shot classification by embedding every axial slice of a given CT image. To compute a similarity score between a CT scan and a given prompt, we take the mean of the cosine similarities between each axial slice and the prompt embedding.

We also perform zero-shot spine fracture detection using the VerSe dataset.^55^ All CTs in the VerSe dataset were evaluated for fracture severity at each vertebral level in the thoracolumbar spine. We reorient and resample the VerSe CTs to match Merlin pretraining. For volumes with fractures, we create a probability map from the fracture grading annotations and sample high-probability locations to obtain representative sub-volumes. We map the Hounsfield unit range −1000:1000 to 0:1 and spatially pad and crop the images to 224 × 224 × 160. For volumes without fractures, we center crop the CTs.

We perform an ablation study where we measure the impact of I3D initialization, staged versus multi-task training with EHR and radiology reports, and splitting the report text with every other batch for finer grain contrastive learning (Figure 2e). We also examine how zero-shot performance varies across the pretraining dataset sizes of 1%, 10%, 20%, 40%, and 100% of the total training set (Figure 2d).

#### Phenotype Classification:

To generate the labels for this task, we group ICD-9 and ICD-10 codes into phenotypes using the PheWAS^57^ phecode mapping. We compare Swin Transformer, ConvNeXt, and ResNet backbone architectures (Figure 3(c)), as well as various model stem hyper-parameters (Figure 3(d)). See section 4.2 for additional details. We report average AUROC and AUPRC using all phenotypes that have more than 20 positive examples in the test set, in order to ensure a meaningful measure of performance. We additionally assess how phenotype classification performance varies across the pretraining dataset sizes of 1%, 10%, 20%, 40%, and 100% of 15,331 total pretraining samples.

Counterfactual analysis (Figure 3(e)) seeks to evaluate whether a model is using expected features during phenotype classification. Models can instead learn shortcuts from spurious correlations in the training data,^81,82,83^ which are easier to learn compared to more subtle physiological features that the model should use. We employ the Latent Shift approach^60^ for counterfactual generation. This method uses a latent variable model, which represents the input image in a low dimensional space and reconstructs the image in its output. The gradient of the Merlin prediction is passed back to the low-dimensional space, where the latent representation is modified such that Merlin’s prediction decreases. This generates a modified image at the output of the latent variable model, which can be observed. The latent variable model regularizes the change in pixels so that the image remains close to the data manifold. The result of the Merlin counterfactual generation process is a new CT volume, which is evaluated by observing how the classifier prediction changes and how the image features driving the classification output are exaggerated or curtailed. We leverage this method to qualitatively verify that the features used during classification are consistent with what we expect the model to have learned. For this evaluation, we identify pleural effusion and splenomegaly as phenotypes with features that are well understood and can be observed in 2D slices. We select the presented volumes such that the phenotype prediction is above the decision boundary determined by AUROC curve analysis, to ensure that we are explaining a positive prediction.

#### Zero-Shot Cross-Modal Retrieval:

In Figure 4(a) we illustrate the procedure for performing zero-shot retrieval. First, we sample a pool of report findings sections. Then, we embed each of the findings sections using the text encoder and the corresponding CT scans using the image encoder. We then compute the cosine similarities between the images and each of the report findings sections. Based on these cosine similarities, we rank the report findings in order of their similarity to each of the images.

To evaluate retrieval performance, we divide the test dataset into non-overlapping pools and within each pool evaluate how often the cosine similarity between corresponding CT images and reports is the highest (Recall@1). To compute the overall score, we average performance across all pools. We compute retrieval performance for surfacing the most similar findings section given a CT image (Image → Findings in Figure 4c) and the most similar CT image given a findings section (Findings → Image in Figure 4c). We compute retrieval performance between images and findings sections on both our internal test dataset (5,137 pairs) and our external dataset (7,000 pairs).

To validate that the model is not learning a shortcut to match images with findings sections, which seems plausible given the length of the findings sections, we also evaluate retrieval between the images and impressions sections. Given that during training, Merlin is only exposed to findings sections from the reports, evaluating on semantically similar, yet distinct impressions sections can provide a measure of generalizability. We also perform this analysis on our internal test dataset and the external dataset (Figure 4c).

Similar to zero-shot classification, we adapt existing 2D baselines (OpenCLIP and BioMedCLIP) for 3D cross-modality retrieval by embedding each axial slice of the CT volumes.

As with zero-shot classification, we perform ablation studies where we examine the impact of I3D initialization, staged versus multi-task training with EHR and radiology reports, and radiology report splitting (Figure 4d). We also perform data scaling experiments that assess how retrieval performance varies across pretraining dataset sizes of 1%, 10%, 20%, 40% and 100% of the full pretraining dataset (Figure 4e).

### 4.5 Adapted Tasks

#### Multi-Disease 5-Year Prediction:

Following previous work,^13^ we choose 6 chronic diseases based on their prevalence and the potential for beneficial interventions following early diagnoses: chronic kidney disease (abbr. CKD; prevalence = 36 million in US^84^), diabetes mellitus (abbr. DM; prevalence = 35 million in US^85^), hypertension (abbr. HTN; prevalence = 120 million in US^86^), ischemic heart disease (abbr. IHD; prevalence = 21 million in US^87^), atherosclerotic cardiovascular disease (abbr. CVD; prevalence = 24 million in US^88^), and osteoporosis (abbr. OST; prevalence = 10 million in US^89^).

To create the labels for this task, we extract disease ICD codes of interest from the EHR. We assign patients to one of 4 categories for each disease based on their ICD codes. Formally, we define *t*_*d*_ to be the time point marking the onset of the disease based on the presence of ICD codes, and *t*_*s*_ to be the time point marking the date of the CT scan. *t*_*a*_ is the time point after a CT scan that defines a window starting with the scan date (*t*_*s*_ to *t*_*a*_), where the presence of a new diagnosis indicates a positive case. We set *t*_*a*_ to 5 years to ensure an adequate time duration for patient followup following the CT imaging as well as providing an adequate duration for intervention in future prospective studies. We define *t*_*h*_ to be the time point marking the last date of record in a patient’s history. With these time points, we classify images into the following categories:

- **Class 0 (Healthy):** The patient is not diagnosed with the specified diseases before their CT scan date (*t*_*s*_) or during the window from *t*_*s*_ to *t*_*a*_. If a patient is diagnosed with the disease, it happens after *t*_*a*_. For patients to be assigned to class 0, they must have 5 years of followup in their EHR history.
- **Class 1 (Progressors):** The patient is not diagnosed with the disease before their CT date, *t*_*s*_, but receives a positive diagnosis between *t*_*s*_ and *t*_*a*_.
- **Class 2 (Already Progressed):** The patient is already diagnosed with the disease before the CT scan date, *t*_*s*_.
- **Class 3 (Censored):** The patient has not been monitored long enough to rule out the disease. In this case, *t*_*h*_ is before *t*_*a*_ and the patient does not receive a positive diagnosis before the end of their EHR history, *t*_*h*_.

For training, we use class 1 examples as positive examples and class 0 examples as negative examples. We fine-tune Merlin on examples that are held out from Merlin pretraining and validation. Furthermore, we do multi-task multi-disease prediction where the fine-tuned Merlin has one head per disease as shown in Figure 10a. Since we train jointly on all the diseases, we mask labels in a batch that are from classes other than 0 and 1 for a specific disease. We use binary cross entropy loss, an AdamW optimizer with a learning rate of 1e-5, an exponential learning rate decay with *γ* = 0.8, and a batch size of 8 on a single A6000 GPU. We use 60%, 20%, 20% train, validation, and test splits, and measure performance in both the low-label regime (10% of training labels with 9–20 positive examples per disease), as well as the medium-label regime (100% of available training labels with 69–136 positive examples per disease). The number of per-disease positive and negative examples are given in Figure 5.

#### Radiology Report Generation:

Figure 8a demonstrates the procedure for adapting Merlin for report generation. We first extract image features from the last hidden layer of Merlin, which has size 7×7 in-plane, 10 out-of-plane, and a feature dimension of 2048. We use a single linear adapter layer to map the features to size 4096. For text generation, we use RadLlama-7B,^42^ a version of Llama2–7B that is fine-tuned on MIMIC^90^ radiology reports and clinical notes. We fine-tune the linear adapter, as well as 5% of RadLlama-7B parameters using low-rank adaptation (LoRA).^91^ We generate the full reports section by section, where we use the following prompt template:


<visual tokens>Generate a radiology report for <organ system>###
<report section>###</s>


For training, we use 8 gradient accumulation steps with a local batch size of 6, giving us a total batchsize of 48. We use an AdamW optimizer with betas=(0.9, 0.999), a learning rate of 1e-4, and a cosine learning rate scheduler with 500 warmup steps and decay to 0 over 500 epochs.

We compare our model performance on report finding section generation versus RadFM^37^ using four metrics: BLEU score^66^ and ROUGE-2,^65^ which primarily assess syntactic similarity; RadGraph-F1,^63^ which assesses findings and modifiers of findings (e.g. location and severity); and BERT score,^64^ a model-based score which assesses semantic similarity. The comparison is conducted both section-by-section and for the full findings sections. Subsequently, we apply qualitative frameworks^92, 93^ to compare our model generated findings to radiologist findings in Figure 8c. This consists of densely annotating individual phrases to be correct, mischaracterized, false positive, or false negative.

#### 3D Semantic Segmentation

To adapt Merlin for segmentation, we add a UNet^94^ decoder and skip connections between the Merlin encoder and the decoder. Each block of the decoder consists of a 3D transpose convolution operation, with a kernel size of 2 and a stride of 2 in each dimension, followed by two 3D convolutions, with kernel sizes of 3 and strides of 1 in each dimension. Each of the two 3D convolution operations is followed by a 3D batchnorm and ReLU activation. We also add skip connections where outputs from the Merlin ResNet blocks are concatenated with outputs from the 3D transpose convolution in the decoder before being passed to the subsequent 3D convolutional layers. For model training, we use both a cross-entropy loss and a Dice loss. We use an AdamW optimizer with betas=(0.9, 0.999) and a learning rate of 5e-5. To mirror pretraining, we segment full volumes of size 224×224×160. We achieve this with 4 gradient accumulation steps and a local batchsize of 1, providing a total batchsize of 4.

For model training and evaluation, we filter body CT scans from the Total Segmentator dataset using the following study types: ct pelvis, ct abdomen-pelvis, ct abdomen, ct thorax, ct thorax-abdomen, and ct thorax-abdomen-pelvis. This gives us 401 scans total. We also select the following organs that appear in abdominal CT: stomach, liver, gallbladder, left kidney, right kidney, spleen, prostate, T12 vertebrae, L1 vertebrae, L2 vertebrae, L3 vertebrae, L4 vertebrae, L5 vertebrae, S1 vertebrae, sacrum, urinary bladder, colon, duodenum, small bowel, and pancreas. We use the official Total Segmentator test split for testing, which has 34 scans after filtering. We split the remaining 367 scans into 80% training examples (293 scans) and 20% validation examples (74 scans). In addition to training with all of the training scans, we simulate the label scarce setting by sampling 10% of training scans randomly.

We compare performance across several model variations using Dice score. We compare Merlin to models with the same architecture but initialized randomly or using ImageNet I3D initialization. We also compare against, Swin UNETR,^68^ an architecture specifically designed for 3D medical image segmentation.

### 4.6 Statistical Analysis

We compute 95% confidence intervals using bootstrapping with 1000 samples with replacement for all experiments except for cross-modal retrieval. For cross-modal retrieval, we divide the test dataset into pools of size N. For each pool, we compute the average retrieval performance for all examples in the pool. We then compute the 95% confidence intervals using the distribution of performances across pools. All p-values that we report are one-sided p-values.

## Supplementary Material

1

## Figures and Tables

**Figure 6:**
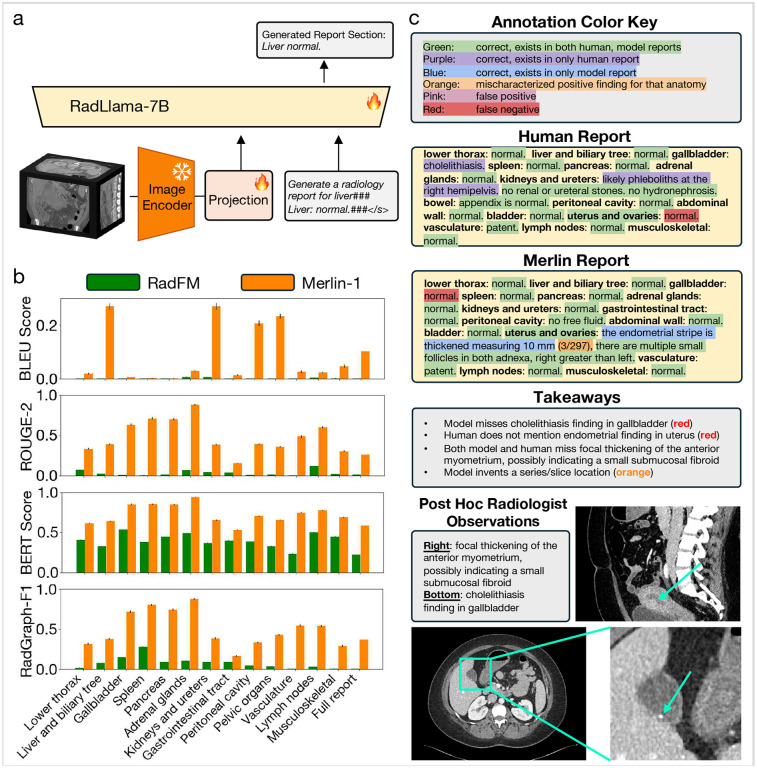
Radiology report generation. (a) To enable report generation, we extract the last hidden layer embeddings from Merlin and modify the dimension of these embeddings using a projection layer. We generate the report section by section and therefore also embed a report section prompt. The resulting vision and language tokens are used as input to a language model to generate a report section. (b) We compare the performance of our model against RadFM, using 4 metrics, across each report section and the full report. (c) We provide a densely annotated example of human and Merlin generated reports. We bold the report section headers in the human and Merlin generated reports. We include “uterus and ovaries” in green, as Merlin needs to deduce the correct pelvic anatomy.

## Footnotes

* Number of job openings on the ACR job board based on accessing the site on May 26, 2024.

† We note that in the clinical context, 3D medical imaging is considered as a subset of cross-sectional imaging where the pixels are isotropic. In this paper, we use the term 3D to align with the terminology used by the machine learning community describing the simultaneous processing of pixels originating from 3 spatial dimensions

‡ Named after the legendary figure said to be able to perceive everything in the present, our model Merlin simultaneously processes the entirety of 3D information in a CT volume.
