# Supplementary material for: Merlin: A Vision Language Foundation Model for 3D Computed Tomography
Source: Res Sq. 2024 Jun 28:rs.3.rs-4546309. Preprint. [Version 1] doi: 10.21203/rs.3.rs-4546309/v1 (PMC11230513; doi:10.21203/rs.3.rs-4546309/v1)
Supplement: 1 [file NIHPPrs4546309V1-supplement-1.pdf]

Supplementary

Split	2D Slices	CTs	Findings Tokens	ICD9 Codes	ICD10 Codes	Patients
Train	6,387,231	15,331	6,036,645	577,998	1,261,561	11,010
Validation	2,099,217	5,060	1,985,925	178,194	379,733	3,644
Test	2,142,061	5,137	2,029,001	197,821	399,986	3,667
Total	10,628,509	25,528	10,051,571	954,013	2,041,280	18,321

Table 1: *Summary of pretraining dataset splits.*

Demographic	Patients (n = 18,321)	Value
**Age**	-	53.8±19.5
**Gender**		
Female	10,254	55.97%
Male	8,065	44.02%
**Self-Reported Race/Ethnicity**		
Non-Hispanic White	8,660	47.27%
Asian	2,673	14.59%
Black	952	5.20%
Hispanic White	515	2.81%
Pacific Islander	294	1.60%
Native American	65	0.35%
Unknown	5,127	27.98%
**Patient Class**		
Inpatient	6,834	37.31%
Emergency Services	6,388	34.87%
Outpatient	2,959	16.16%
Observation	1,452	7.93%

Table 2: *Internal dataset characteristics.* The age value is provided as mean ± standard deviation. All other values are provided as percentages of the total patients (n = 18,321).

Demographic	Patients (n = 5,804)	Value
**Age**	-	61.4±16.5
**Gender**		
Female	2,982	51.38%
Male	2,822	48.62%
**Self-Reported Race/Ethnicity**		
Non-Hispanic White	4,576	78.84%
Black	270	4.65%
Hispanic White	198	2.76%
Asian	87	1.50%
Native American	31	0.53%
Pacific Islander	4	0.07%
Unknown	602	10.37%

Table 3: *External dataset characteristics.* The age value is provided as mean ± standard deviation. All other values are provided as percentages of the total patients (n = 5,804).

Encoder	*Init*	*Labels*	*Stem* KS$_z$/ Stride$_z$	All Phecodes N=691, Prev=3.1%		Phecodes w/ AUC		Upper Quartile by Prevalence N=173; Prev=8.7%		Lower Quartile by Prevalence N=173, Prev=0.6%	
				AUROC	AUPRC	>0.85	>0.9	AUROC	AUPRC	AUROC	AUPRC
SwinUNETR	MAE	EHR	4/4	.736	.088	52	8	.734	.207	.738	.029
ConvNeXt-T	I3D	EHR	7/2	.768	.106	115	20	.765	.239	.764	.035
ConvNeXt-S	I3D	EHR	7/2	.761	.102	105	14	.760	.234	.750	.031
ConvNeXt-B	I3D	EHR	7/2	.773	.110	132	28	.768	.244	.766	.036
ConvNeXt-B*	I3D	EHR	3/2	.789	.131	180	46	.784	.270	.781	.052
ResNet50	I3D	EHR	3/1	.798	.137	226	72	.787	.280	.797	.049
ResNet152	I3D	EHR	7/2	.798	.135	221	65	.785	.272	.796	.050
↓	I3D	EHR	3/2	.798	.136	221	74	.788	.275	.792	.049
	I3D	EHR	3/1	.801	.140	235	79	.789	.279	.801	**.054**
(Merlin)	I3D	MTL	3/1	**.812**	**.142**	**259**	**93**	**.804**	**.290**	**.808**	.050

Table 4: *Phenotype classification.* We only include phenotypes that have more than 20 positive examples in the test set in order to ensure a meaningful measure of performance. * in ConvNext-B* indicates that instead of inflating the z dimension to a size equal to the 2D kernel height and width of 7, the kernel is inflated to a depth of 3.

Init	*Labels*	*Split Text*	*Stem* KS$_z$/ Stride$_z$	All Phecodes N=691, Prev=3.1%		Phecodes w/ AUC		Upper Quartile by Prevalence N=173; Prev=8.7%		Lower Quartile by Prevalence N=173, Prev=0.6%	
				AUROC	AUPRC	>0.85	>0.9	AUROC	AUPRC	AUROC	AUPRC
I3D	Staged	✗	3/1	.804	.145	240	84	.792	.287	.798	.056
I3D	Staged	✓	3/1	.807	.146	249	87	.795	.291	.801	.056
I3D	MTL	✗	3/1	**.814**	**.153**	**267**	**103**	**.806**	**.302**	.807	**.058**
Rand	MTL	✓	3/1	.786	.124	117	46	.778	.261	.779	.042
I3D	MTL	✓	3/1	.812	.142	259	93	.804	.290	**.808**	.050

Table 5: *Phenotype classification ablation study.* We perform ablation studies where we examine the impact of I3D initialization, staged training versus multi-task learning (MTL) with EHR and radiology reports, and splitting the report text with every other batch for finer grain contrastive learning.

Method	**Average F1 Score**
OpenCLIP (Internal)	.276 [.262, .288]
BioMedCLIP (Internal)	.285 [.274, .295]
Merlin (Internal)	.741 [.727, .755]
Merlin (External)	.647 [.607, .678]
Merlin (VerSe)	.767 [.630, .867]

Table 6: *Zero-shot classification.* The internal and external numbers (first 4 rows) represent averages over the 30 findings for the internal clinical dataset and the external clinical dataset respectively. The bottom row represents F1 score for zero-shot classification of vertebral fractures on the VerSe dataset.

Init	*Labels*	*Split Text*	**Average F1 Score**
I3D	Report	✓	.730 [.714, .744]
I3D	Staged	✗	.669 [.653, .683]
I3D	Staged	✓	.735 [.719, .748]
I3D	MTL	✗	.656 [.640, .671]
Rand	MTL	✓	.698 [.681, .711]
I3D	MTL	✓	**.741 [.727, .755]**

Table 7: *Zero-shot classification ablation study.* We measure zero-shot performance as we vary parameters of I3D versus random initialization, staged training versus multi-task learning (MTL) with EHR and radiology reports, and training with the full findings sections versus using radiology report splitting.

Task	*Method*	**Recall@1**			**Recall@8**		
		N=32	N=64	N=128	N=32	N=64	N=128
Img→F	OpenCLIP	.030	.016	.009	.243	.125	.062
	BioMedCLIP	.040	.021	.010	.298	.156	.083
	Merlin	**.780**	**.696**	**.608**	**.989**	**.968**	**.927**
F→Img	OpenCLIP	.033	.017	.009	.250	.125	.061
	BioMedCLIP	.044	.021	.012	.306	.156	.079
	Merlin	**.776**	**.687**	**.594**	**.988**	**.965**	**.920**
Img→I	OpenCLIP	.030	.016	.010	.256	.128	.061
	BioMedCLIP	.036	.017	.009	.273	.141	.073
	Merlin	**.352**	**.253**	**.174**	**.796**	**.663**	**.532**
I→Img	OpenCLIP	.032	.017	.008	.252	.126	.064
	BioMedCLIP	.046	.024	.012	.322	.169	.081
	Merlin	**.384**	**.277**	**.194**	**.854**	**.706**	**.564**

Table 8: *Cross-modality retrieval.* We compare performance of OpenCLIP, BioMedCLIP, and Merlin across several settings: retrieving the correct findings section given an image (Img → F), retrieving the correct image given a findings section (F → Img), retrieving the correct impressions section given an image (Img → I), and retrieving the correct image given an impressions section (I → Img). We perform retrieval within pools of sizes N=32, N=64, and N=128.

Task	*Init*	*Labels*	*Split*	**Recall@1**			**Recall@8**		
			Text	N=32	N=64	N=128	N=32	N=64	N=128
Img→F	I3D	Report	✓	.778	.692	.598	**.989**	.967	.921
	I3D	Staged	✗	.654	.547	.449	.967	.920	.844
	I3D	Staged	✓	.672	.561	.457	.972	.925	.848
	I3D	MTL	✗	**.812**	**.726**	**.639**	.988	**.969**	**.937**
	Rand	MTL	✓	.690	.583	.468	.978	.937	.867
	I3D	MTL	✓	.780	.696	.608	**.989**	.968	.927
F→Img	I3D	Report	✓	.775	.686	.584	.991	**.969**	.921
	I3D	Staged	✗	.646	.539	.434	.965	.913	.841
	I3D	Staged	✓	.664	.555	.445	.970	.923	.841
	I3D	MTL	✗	**.801**	**.718**	**.626**	**.988**	.968	**.933**
	Rand	MTL	✓	.683	.571	.455	.980	.940	.869
	I3D	MTL	✓	.776	.687	.594	**.988**	.965	.920
Img→I	I3D	Report	✓	.364	.265	.187	**.812**	**.681**	**.549**
	I3D	Staged	✗	.307	.220	.159	.737	.590	.449
	I3D	Staged	✓	.328	.228	.163	.780	.634	.500
	I3D	MTL	✗	**.372**	**.275**	**.196**	.799	.667	.543
	Rand	MTL	✓	.288	.202	.131	.740	.592	.453
	I3D	MTL	✓	.352	.253	.174	.796	.663	.532
I→Img	I3D	Report	✓	.382	.274	.192	.850	**.709**	**.574**
	I3D	Staged	✗	.324	.234	.161	.770	.616	.490
	I3D	Staged	✓	.348	.246	.168	.811	.672	.523
	I3D	MTL	✗	**.400**	**.294**	**.216**	.817	.698	.568
	Rand	MTL	✓	.289	.200	.127	.779	.613	.460
	I3D	MTL	✓	.384	.277	.194	**.854**	.706	.564

Table 9: *Cross-modality retrieval ablation study.* We compare retrieval performance across three axes of weight initializations, methods for incorporating EHR and radiology reports into training, and splitting or using the full findings during training.

Encoder	*Init*	*Labels*	*%Tr*	**CKD**	**DM**	**HTN**	**IHD**	**CVD**	**OST**	**Average**
			10%	14/46	9/46	11/34	9/49	20/52	10/47	
			100%	90/513	80/474	111/404	69/518	136/504	68/527	
Swin Transformer	-	-	10%	.46 [.40, .50]	.70 [.66, .75]	.43 [.39, .48]	.53 [.49, .57]	.53 [.50, .57]	.56 [.51, .61]	.54 [.52, .55]
ResNet152	-	-	10%	.53 [.48, .58]	.66 [.61, .71]	.60 [.55, .64]	.49 [.45, .54]	.58 [.54, .62]	.50 [.44, .56]	.56 [.54, .58]
↓	I3D	-	10%	.72 [.67, .76]	**.72 [.67, .76]**	.67 [.63, .71]	.67 [.63, .71]	.67 [.64, .71]	.66 [.61, .71]	.68 [.67, .70]
	I3D	EHR	10%	.58 [.53, .63]	.64 [.59, .69]	.47 [.42, .51]	**.75 [.71, .78]**	.69 [.65, .72]	.62 [.57, .67]	.62 [.60, .64]
(Merlin)	I3D	MTL	10%	**.74 [.70, .78]**	.70 [.66, .75]	**.69 [.65, .73]**	.70 [.67, .74]	**.73 [.69, .76]**	**.69 [.65, .73]**	**.71 [.69, .72]**
Swin Transformer	-	-	100%	.55 [.50, .59]	.73 [.68, .77]	.61 [.57, .65]	.52 [.48, .56]	.54 [.49, .57]	.60 [.55, .66]	.59 [.57, .61]
ResNet152	-	-	100%	.63 [.58, .67]	**.74 [.69, .78]**	.65 [.61, .69]	.65 [.61, .69]	.57 [.54, .61]	.60 [.55, .66]	.64 [.62, .66]
↓	I3D	-	100%	.74 [.70, .77]	**.74 [.70, .78]**	.71 [.67, .75]	.68 [.64, .72]	.68 [.64, .71]	.74 [.69, .78]	.71 [.70, .73]
	I3D	EHR	100%	.76 [.73, .80]	.72 [.68, .76]	.74 [.70, .77]	.74 [.70, .78]	.73 [.69, .76]	.68 [.64, .73]	.73 [.71, .74]
(Merlin)	I3D	MTL	100%	**.77 [.74, .81]**	.72 [.68, .76]	**.75 [.72, .79]**	**.76 [.72, .79]**	**.74 [.71, .77]**	**.80 [.76, .84]**	**.76 [.74, .77]**

Table 10: *Multi-disease 5-year prediction.* We fine-tune Merlin for 5-year disease prediction. All data used in this evaluation, including train, val, and test splits, are held out from pretraining.

	**BLEU ↑**		**ROUGE-2 ↑**		**BERT ↑**		**RadGraph-F1 ↑**	
Section	RadFM	Merlin	RadFM	Merlin	RadFM	Merlin	RadFM	Merlin
Lower thorax	.001	.019	.070	.332	.406	.615	.020	.319
Liver and biliary tree	.001	.269	.025	.389	.328	.641	.080	.380
Gallbladder	.000	.006	.006	.632	.534	.851	.152	.721
Spleen	.000	.002	.004	.710	.382	.853	.283	.805
Pancreas	.000	.001	.010	.700	.447	.849	.091	.748
Adrenal glands	.006	.030	.067	.882	.490	.942	.106	.879
Kidneys and ureters	.005	.269	.040	.385	.368	.654	.091	.387
Gastrointestinal tract	.001	.013	.037	.152	.398	.531	.092	.167
Peritoneal cavity	.000	.206	.005	.390	.387	.702	.050	.335
Pelvic organs	.000	.233	.009	.358	.328	.656	.036	.432
Vasculature	.000	.026	.004	.485	.232	.748	.006	.548
Lymph nodes	.003	.023	.119	.601	.502	.775	.031	.542
Musculoskeletal	.001	.046	.018	.303	.449	.689	.008	.293
Full report	.000	.102	.011	.262	.224	.588	.008	.293

Table 11: *Radiology report generation.* We compare Merlin and RadFM for generating radiology report sections corresponding to various anatomies, as well as the full findings.

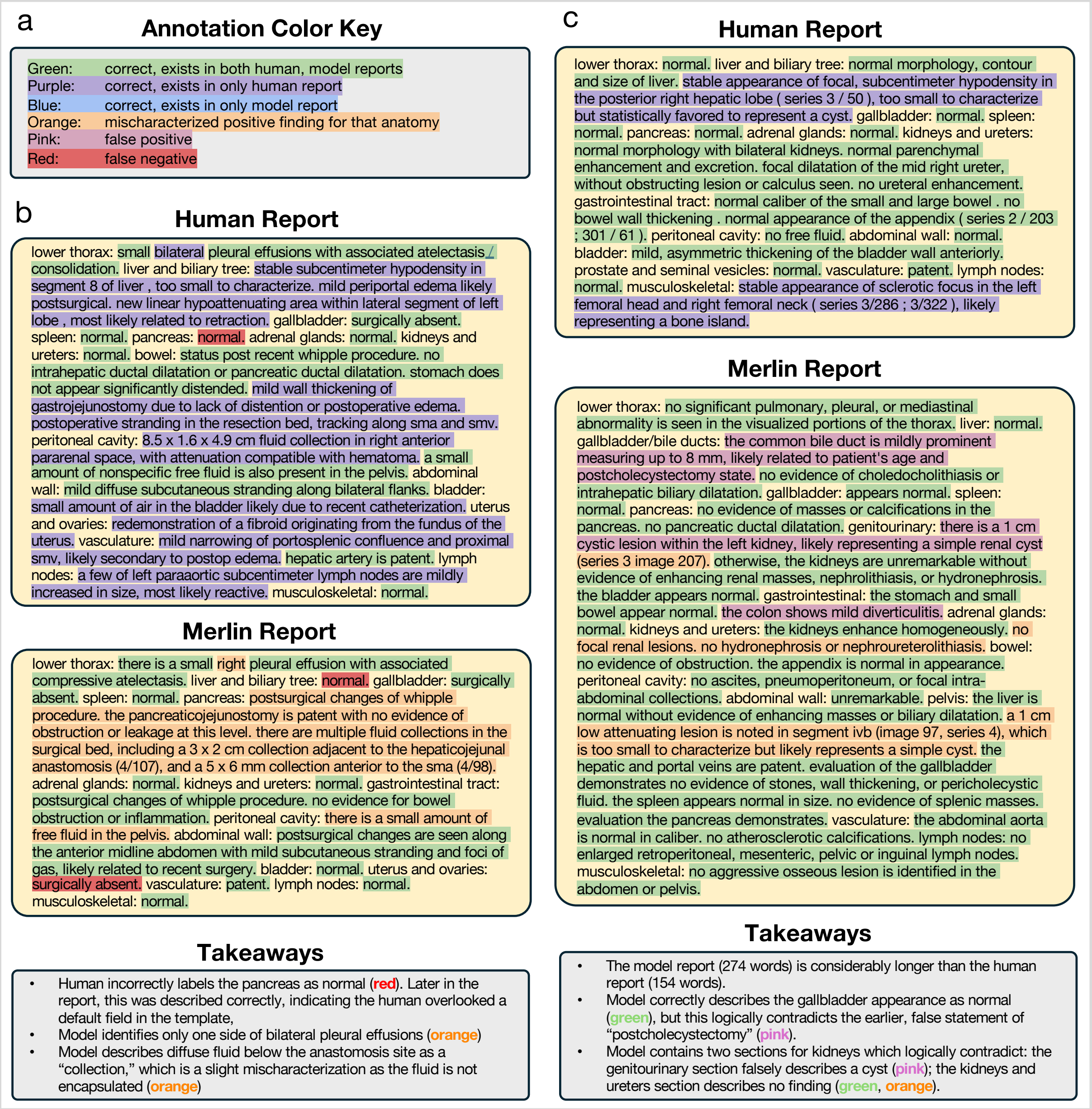

Figure 8: *Radiology report generation.* (a) As shown in the annotation color key, we annotate individual phrases to be correct, mischaracterized, false positive, or false negative. (b - c) We provide dense annotations of two sets of human and Merlin generated reports.
